# Brief Report: The Genetic Profile of Rheumatoid Factor–Positive Polyarticular Juvenile Idiopathic Arthritis Resembles That of Adult Rheumatoid Arthritis

**DOI:** 10.1002/art.40443

**Published:** 2018-04-21

**Authors:** Anne Hinks, Miranda C. Marion, Joanna Cobb, Mary E. Comeau, Marc Sudman, Hannah C. Ainsworth, John Bowes, Juvenile Idiopathic Arthritis Consortium for Immunochip, Mara L. Becker, John F. Bohnsack, Johannes‐Peter Haas, Daniel J. Lovell, Elizabeth D. Mellins, J. Lee Nelson, Ellen Nordal, Marilynn Punaro, Ann M. Reed, Carlos D. Rose, Alan M. Rosenberg, Marite Rygg, Samantha L. Smith, Anne M. Stevens, Vibeke Videm, Carol A. Wallace, Lucy R. Wedderburn, Annie Yarwood, Rae S. M. Yeung, Carl D. Langefeld, Susan D. Thompson, Wendy Thomson, Sampath Prahalad

**Affiliations:** ^1^ University of Manchester Manchester UK; ^2^ Wake Forest University School of Medicine Winston‐Salem North Carolina; ^3^ University of Manchester and Central Manchester University Hospitals NHS Foundation Trust Manchester UK; ^4^ Cincinnati Children's Hospital Medical Center Cincinnati Ohio; ^5^ Children's Mercy Kansas City Kansas City Missouri; ^6^ University of Utah Salt Lake City; ^7^ German Centre for Pediatric and Adolescent Rheumatology Garmisch‐Partenkirchen Germany; ^8^ Stanford University Stanford California; ^9^ Fred Hutchinson Cancer Research Center and University of Washington Seattle; ^10^ University Hospital of North Norway and UIT The Arctic University of Norway Tromsø Norway; ^11^ Arthritis Clinic Texas Scottish Rite Hospital for Children and University of Texas Southwestern Medical Center Dallas; ^12^ Duke University School of Medicine Durham North Carolina; ^13^ DuPont Children's Hospital Wilmington Delaware; ^14^ University of Saskatchewan Saskatoon Saskatchewan Canada; ^15^ Norwegian University of Science and Technology and St. Olav's University Hospital Trondheim Norway; ^16^ Seattle Children's Research Institute and University of Washington Seattle; ^17^ Seattle Children's Hospital and Research Institute Seattle Washington; ^18^ University College London and NIHR Great Ormond Street Hospital Biomedical Research Centre London UK; ^19^ The Hospital for Sick Children and University of Toronto Toronto Ontario Canada; ^20^ Emory University School of Medicine and Children's Healthcare of Atlanta Atlanta Georgia

## Abstract

**Objective:**

Juvenile idiopathic arthritis (JIA) comprises 7 heterogeneous categories of chronic childhood arthritides. Approximately 5% of children with JIA have rheumatoid factor (RF)–positive arthritis, which phenotypically resembles adult rheumatoid arthritis (RA). Our objective was to compare and contrast the genetics of RF‐positive polyarticular JIA with those of RA and selected other JIA categories, to more fully understand the pathophysiologic relationships of inflammatory arthropathies.

**Methods:**

Patients with RF‐positive polyarticular JIA (n = 340) and controls (n = 14,412) were genotyped using the Immunochip array. Single‐nucleotide polymorphisms were tested for association using a logistic regression model adjusting for admixture proportions. We calculated weighted genetic risk scores (wGRS) of reported RA and JIA risk loci, and we compared the ability of these wGRS to predict RF‐positive polyarticular JIA.

**Results:**

As expected, the HLA region was strongly associated with RF‐positive polyarticular JIA (*P* = 5.51 × 10^−31^). Nineteen of 44 RA risk loci and 6 of 27 oligoarticular/RF‐negative polyarticular JIA risk loci were associated with RF‐positive polyarticular JIA (*P* < 0.05). The RA wGRS predicted RF‐positive polyarticular JIA (area under the curve [AUC] 0.71) better than did the oligoarticular/RF‐negative polyarticular JIA wGRS (AUC 0.59). The genetic profile of patients with RF‐positive polyarticular JIA was more similar to that of RA patients with age at onset 16–29 years than to that of RA patients with age at onset ≥70 years.

**Conclusion:**

RF‐positive polyarticular JIA is genetically more similar to adult RA than to the most common JIA categories and thus appears to be a childhood‐onset presentation of autoantibody‐positive RA. These findings suggest common disease mechanisms, which could lead to novel therapeutic targets and shared treatment strategies.

Juvenile idiopathic arthritis (JIA) is a heterogeneous collection of chronic arthropathies with distinct clinical and laboratory features, but all manifest with arthritis in one or more joints and present before the 16th birthday. The International League of Associations for Rheumatology (ILAR) criteria for JIA recognize 7 JIA categories 1. There is robust evidence for genetic factors conferring susceptibility to all forms of JIA 2. Without a clearer understanding of the genetic similarities and distinctions, the clinically different categories must be studied separately. Unfortunately, this stratification results in smaller sample sizes and reduced power to detect association. Thus, the JIA Consortium for Immunochip was formed with the intent to bring together the large sample sizes required for investigation of the rarer JIA categories. A full list of affiliations for consortia appears in Supplementary Information, available on the *Arthritis & Rheumatology* web site at http://onlinelibrary.wiley.com/doi/10.1002/art.40443/abstract. The Immunochip is a custom microarray designed by the Immunochip Consortium to fine‐map autoimmune disease–associated loci from 11 autoimmune phenotypes including adult rheumatoid arthritis (RA) 3. The Immunochip assays 196,524 variants representing ~186 loci, including dense coverage of the major histocompatibility complex region. Investigation of children with the most common categories of JIA, oligoarticular and rheumatoid factor (RF)–negative polyarticular JIA, which comprise ~70% of all cases in children of European descent, resulted in the identification of 17 loci associated with JIA at genome‐wide levels of significance. In addition, 11 loci showed suggestive evidence of association 4.

Approximately 5% of children with JIA demonstrate the presence of RF and antibodies directed against citrullinated peptides, such as anti–cyclic citrullinated peptide (anti‐CCP) antibodies, which are characteristic biomarkers observed in adults with seropositive RA. These children and young people tend to present at a later age at onset than those with oligoarticular or RF‐negative polyarticular JIA, and often tend to have erosive disease with worse long‐term outcomes. Thus, children with RF‐positive polyarticular JIA phenotypically resemble adults with RA and could be considered to have childhood‐onset RA. In contrast to the robust genetic studies that include large cohorts of patients with RA and oligoarticular/RF‐negative polyarticular JIA, studies of children with RF‐positive polyarticular JIA have been limited to small‐scale candidate gene studies. These include investigations of association with the shared epitope encoding HLA–DRB1 alleles as well as several candidate loci associated with RA 5, 6. To date, a systematic analysis of genetic risk for RF‐positive polyarticular JIA has not been completed, largely due to the lack of sufficiently sized cohorts.

To progress beyond this limitation in cohort size and also advance the understanding of RF‐positive polyarticular JIA, we have used the Immunochip to compare and contrast the genetics of RF‐positive polyarticular JIA to other categories of JIA and RA. This may provide a greater understanding of the genetic architecture of RF‐positive polyarticular JIA.

## Patients and methods

All JIA patients had a diagnosis of polyarticular JIA according to the ILAR classification criteria 1 and were positive for RF and/or anti‐CCP antibodies. The ILAR criteria do not include any recommendation for anti‐CCP testing; therefore, anti‐CCP is not routinely tested for in pediatric rheumatology cohorts. We do have anti‐CCP data on 73 subjects (~20%). Of those tested, the prevalence of anti‐CCP positivity is 79%. Among patients who were RF positive, 78% were also positive for anti‐CCP, which is comparable to the value of ~59% reported in the literature for patients with RF‐positive polyarticular JIA 7. Cases were ascertained at institutions in the US, UK, Germany, Canada, and Norway. Genotyping was performed using the Illumina Immunochip genotyping array. There were 421 patients with RF‐positive polyarticular JIA and 16,403 controls before quality control. Standard single‐nucleotide polymorphism (SNP) genotyping and sample quality control were performed as previously described in the Immunochip studies of other JIA categories 4, 8. Details of cohorts can be found in Supplementary Information, http://onlinelibrary.wiley.com/doi/10.1002/art.40443/abstract.

For comparison with groups of RA patients with different ages at onset, RA patients in the UK who had been genotyped on the Immunochip array were available from a cohort described previously 9. RA patients were selected if they fell into 2 categories of age at onset, those with early‐onset RA (ages 16–29 years; n = 370) and those with later‐onset RA (ages ≥70 years; n = 259). In total, 8,675 controls from the RA cohort overlapped with the UK controls used for the JIA cohorts. To preserve independence, these controls were randomly split into 2 groups (see Supplementary Table 1, http://onlinelibrary.wiley.com/doi/10.1002/art.40443/abstract).

To test for SNP association with RF‐positive polyarticular JIA, a logistic regression model was computed using Caucasian admixture proportions calculated by the program ADMIXTURE 10 as covariates. The additive genetic model was used for the primary analysis unless there was significant departure from additivity, whereupon the most associated genetic model was used. For markers on the X‐chromosome, the logistic model was stratified by sex and inference was based on the resulting weighted inverse normal meta‐analysis. Imputation of SNP genotypes was completed using IMPUTE2 with the 1000 Genomes Phase 1 integrated reference panel 11. To test for association with the imputed data, a logistic regression model with admixture adjustment was computed on the imputed allele dosage. Only SNPs that passed standard imputation quality control and had information score >0.5 and confidence score >0.9 were considered for association analysis. For each region we reported the strongest associated genotyped SNP. If there was an imputed SNP that showed stronger association than the genotyped SNP, then both SNPs were reported; imputed SNPs required at least 2 SNPs in strong linkage disequilibrium (LD) to also exhibit association. Regional plots of association were computed using LocusZoom 12.

The 45 non‐HLA risk loci associated with RA using the Immunochip 9 and the 27 oligoarticular/RF‐negative polyarticular JIA non‐HLA risk loci 4 were assessed to determine if they were also associated with RF‐positive polyarticular JIA in our cohort. Two weighted genetic risk scores (wGRS) were calculated. The first used the RA risk loci 9 and the second used the oligoarticular/RF‐negative polyarticular JIA risk loci 4. The RA wGRS analysis started with the 46 SNPs (including HLA) (*P* < 5 × 10^−8^) associated with RA as reported by Eyre et al 9. However, no proxies (r^2^ > 0.8) were available for rs13397 at *IRAK1*, rs2240336 at *PADI4*, rs39984 at *GIN1*, or rs10683701 at *KIF5A*; therefore, there were 42 SNPs in the wGRS. The HLA region was captured through the HLA–DRB1 tag SNP rs660895 13.

The JIA wGRS analysis started with the 28 SNPs (including HLA) (*P* < 1 × 10^−6^) associated with oligoarticular/RF‐negative polyarticular JIA as reported by Hinks et al 4. However, no proxies were available for rs7909519 at *IL2RA*, rs2266959 at *UBE2L3*, and rs7069750 at *FAS*, so the final number of SNPs in the wGRS was 25. The HLA association was captured using the top SNP (rs7775055) in the region.

To calculate the wGRS for an individual, the natural log of the reported odds ratio was multiplied by the number of risk alleles for each SNP and summed. Individuals with missing genotypes were assigned (imputed) a score based on the expectation from the allele frequency and assuming Hardy‐Weinberg equilibrium. Logistic regression was used to compare each wGRS between patients and controls. In addition, receiver operating characteristic (ROC) curves defined by the sensitivity and specificity of each wGRS were generated, and the area under the curve (AUC) was calculated. The GRS analysis did not include the imputed genotype data. Analysis was performed using Stata software, version 13.1 (StataCorp). We tested whether there was a difference between the areas under the 2 ROC curves using DeLong's method as implemented in SAS software (SAS Institute).

## Results

After quality control there were 340 patients with RF‐positive polyarticular JIA (mean ± SD age at onset 10.2 ± 4.2 years) and 14,412 controls (Table 1). For the X‐chromosome analysis, there were 292 female patients, 8,002 female controls, 48 male patients, and 6,410 male controls.

**Table 1:** Study populations of patients with rheumatoid factor–positive polyarticular juvenile idiopathic arthritis and controls before and after quality control

Population	Before quality control		After quality control	
	Patients	Controls	Patients	Controls
US	272	5,985	222	4,408
UK	104	8,940	94	8,579
Germany	15	489	1	480
Norway	14	989	13	945
Canada	16	–	10	–
Total	421	16,403	340	14,412

Despite the modest sample size, association with the HLA region was identified, with the most significant association at rs3129769, near HLA–DRB1 (*P* = 5.51 × 10^−31^), a SNP in strong LD (r^2^ = 0.88) with the HLA–DRB1 SNP reported in RA (rs660895; *P* = 2.14 × 10^−29^). These SNPs are tagging the HLA–DRB1*0401 classic allele 14. There was no significant association of the most associated SNP in the HLA region reported in the oligoarticular/RF‐negative polyarticular JIA Immunochip study, rs7775055 (*P* = 0.08).

The most significantly associated loci identified in the oligoarticular/RF‐negative polyarticular JIA and RA Immunochip study were assessed for association with RF‐positive polyarticular JIA. Of the 27 non‐HLA SNPs most strongly associated with oligoarticular/RF‐negative polyarticular JIA 4, 6 showed evidence for association with RF‐positive polyarticular JIA (*P* < 0.05) (see Supplementary Table 2, http://onlinelibrary.wiley.com/doi/10.1002/art.40443/abstract). Of the 44 SNPs (not including HLA and *KIF5A* regions, the latter being a deletion polymorphism and not analyzed in this study) most strongly associated with RA 9, 19 showed evidence for association with RF‐positive polyarticular JIA (*P* < 0.05) (see Supplementary Table 3, http://onlinelibrary.wiley.com/doi/10.1002/art.40443/abstract).

**Figure 1:**
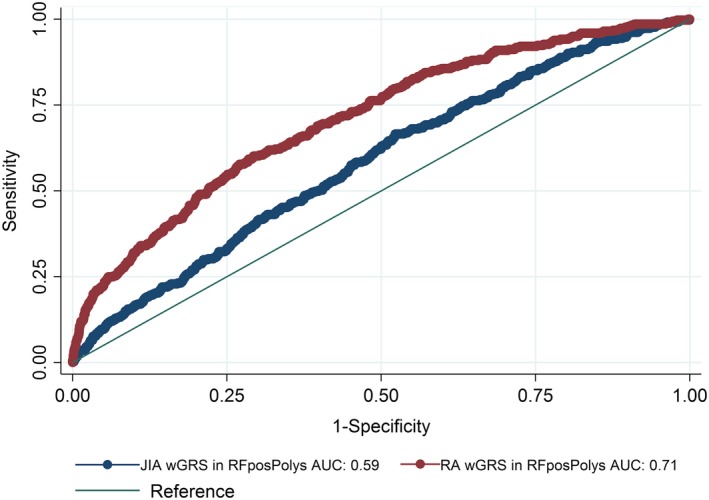
Comparison of the weighted genetic risk score (wGRS) generated using loci associated with the highest risk of rheumatoid arthritis (RA) with the wGRS generated using loci associated with the highest risk of oligoarticular/rheumatoid factor (RF)–negative polyarticular juvenile idiopathic arthritis (JIA) for the purpose of predicting cases of RF‐positive polyarticular JIA (RFposPolys). AUC = area under the curve.

**Figure 2:**
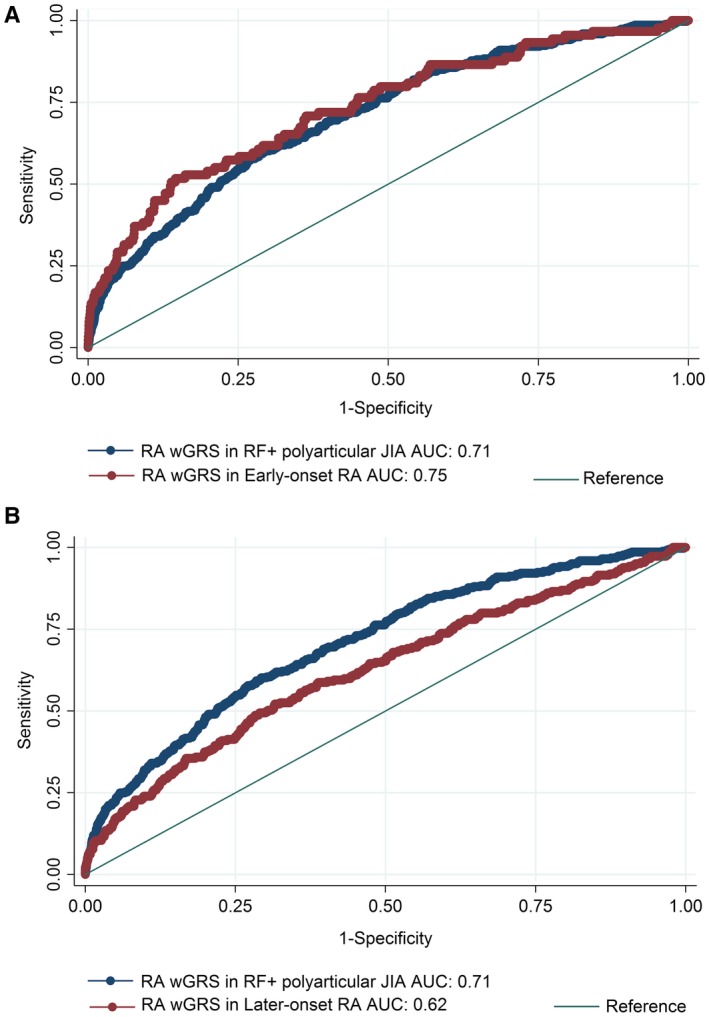
**A,** Comparison of the ability of the wGRS generated using loci associated with the highest risk of RA to predict cases of RF‐positive polyarticular JIA with the ability of the same wGRS to predict cases of early‐onset RA (ages 16–29 years). **B,** Comparison of the ability of the wGRS generated using loci associated with the highest risk of RA to predict cases of RF‐positive polyarticular JIA with the ability of the same wGRS to predict cases of later‐onset RA (age ≥70 years). See Figure 1 for definitions.

The wGRS generated using the top RA loci was compared with the wGRS generated using the top oligoarticular/RF‐negative polyarticular JIA loci to see which best predicted cases of RF‐positive polyarticular JIA compared to controls. The wGRS generated using the top RA loci from Eyre et al 9 significantly improved prediction of cases of RF‐positive polyarticular JIA compared to the wGRS generated using the top oligoarticular/RF‐negative polyarticular JIA loci (AUC 0.71 versus AUC 0.59, respectively; *P* = 8.26 × 10^−33^) (Figure 1). The RA wGRS showed comparable prediction of cases of RF‐positive polyarticular JIA and cases of early‐onset RA (AUC 0.71 versus AUC 0.75, respectively; *P* = 0.25) (Figure 2A) but was less effective at predicting later‐onset RA compared to predicting RF‐positive polyarticular JIA (AUC 0.62 versus AUC 0.71, respectively; *P* = 1.65 × 10^−5^) (Figure 2B). This suggests that the genetic profile of patients with RF‐positive polyarticular JIA is more similar to that of younger RA patients than to that of older RA patients.

No region outside the HLA region reached genome‐wide significance; however, 13 regions had suggestive association (*P* < 1 × 10^−4^). Imputed SNP results were included when the imputed SNP had a better imputed *P* value than the most significant directly genotyped SNP in the region (see Supplementary Table 4 and Supplementary Figures 1 and 2, http://onlinelibrary.wiley.com/doi/10.1002/art.40443/abstract). Supplementary Table 4 denotes imputed SNPs with a “b” superscript. Of the 13 regions most strongly associated with RF‐positive polyarticular JIA, 5 contained SNPs (or SNPs in LD, r^2^ > 0.8) with some previous evidence for association with RA 9.

## Discussion

This represents the largest genetic study of RF‐positive polyarticular JIA to date. We provide evidence that this uncommon category of JIA, which is phenotypically similar to adult seropositive RA, is also genetically more similar to adult RA than to the most common JIA categories, which lack the characteristic biomarkers (RF and anti‐CCP). The results of the wGRS analysis generated from the top RA‐associated loci predicted RF‐positive polyarticular JIA case–control status better than did the wGRS generated from the oligoarticular/RF‐negative polyarticular JIA top hits.

We investigated whether any of the previously associated RA loci 9 or oligoarticular/RF‐negative polyarticular JIA loci 4 showed evidence for association with RF‐positive polyarticular JIA. Nineteen of the 44 SNPs reaching genome‐wide significance thresholds with RA show evidence for association with RF‐positive polyarticular JIA (*P* < 0.05). There appears to be less overlap with the oligoarticular/RF‐negative polyarticular JIA loci since only 6 of the 27 oligoarticular/RF‐negative polyarticular JIA SNPs show evidence for association with RF‐positive polyarticular JIA. Formal testing for a difference in the 2 proportions using the likelihood ratio test yielded suggestive but not statistically significant results (*P* = 0.0676).

As might be expected, the most significant association was within the HLA region, and the SNP is in strong LD (r^2^ = 0.88) with the most associated HLA SNP in RA. We have previously reported the HLA associations for all the categories of JIA 8 and found that RF‐positive polyarticular JIA has distinct HLA associations compared to the other categories of JIA. The HLA–DRB1 amino acid position 13 is most strongly associated with RF‐positive polyarticular JIA, with a histidine residue driving the association. This is the same HLA association as found in RA 8, 13. A glycine residue at this same amino acid position drives the association in oligoarticular/RF‐negative polyarticular JIA. This supports separation of RF‐positive polyarticular JIA from the other JIA categories and confirms that RF‐positive polyarticular JIA is more similar to RA than to other JIA categories 8.

Other than the HLA region, we were unable to identify novel loci meeting genome‐wide levels of significance. This may be expected, as despite being the largest genetic study to date for RF‐positive polyarticular JIA, our study is still relatively underpowered to detect odds ratios of ~1.1–1.2, as are often observed in autoimmune diseases. We have identified 13 regions showing association at a significance level of *P* < 1 × 10^−4^, which will need validation in an independent cohort to confirm. The strongest non‐HLA association for RF‐positive polyarticular JIA was rs9610687, which lies upstream of the *RAC2* gene. Mutations within *RAC2* are associated with neutrophil immunodeficiency syndrome. Polymorphisms within the *IL2RB* gene, close to *RAC2*, have previously been associated with oligoarticular/RF‐negative polyarticular JIA 4 and with RA 9. However, the oligoarticular/RF‐negative polyarticular JIA–associated SNP (rs2284033) is ~500 kb from the RF‐positive polyarticular JIA–associated SNP. The oligoarticular/RF‐negative polyarticular JIA–associated SNP in *IL2RB* was not significantly associated with RF‐positive polyarticular JIA (*P* = 0.70). In RA the most associated SNP (rs3218251) in this region again lies in the *IL2RB* gene, and this SNP is not in LD with the oligoarticular/RF‐negative polyarticular JIA–associated SNP.

Although this study has numerous important findings, there are some important limitations. First, the RA patients included in the wGRS analysis had a mixture of both seronegative and seropositive disease (although the largest proportion were seropositive [68% anti‐CCP positive]), potentially diluting or masking effect sizes. Second, the UK RA patients and controls included in these analyses are part of the RA Immunochip study by Eyre et al 9, and this lack of independence could artificially inflate the predictive ability of the wGRS. A more recent genetic study in RA reported by Okada et al 15 identified 101 genetic regions associated with RA. Many of these regions were not covered on the Immunochip array and so it was not possible to use these in the wGRS analysis 9.

The current ILAR classification criteria 1 are based on clinical features and family history, and it is not always straightforward to assign children to a category. In addition, there still remains heterogeneity, especially in terms of prognosis, between and within the categories of JIA. In time, clear delineation of the genetics of JIA categories may contribute to a more refined classification system. While it has been recognized for many years that RF‐positive polyarticular JIA is clinically and serologically similar to adult RA, there have been no systematic investigations of possible genetic overlap between these phenotypes of inflammatory arthritis. One reason for this is that several JIA categories are rare, and large‐scale international collaborations such as this, and the one established for systemic‐onset JIA, another rare category 16, are necessary to build up sample sizes for genetic studies of these phenotypes.

We have now shown that RF‐positive polyarticular JIA is genetically more similar to adult RA than to the oligoarticular/RF‐negative polyarticular JIA categories. Demonstrating that RF‐positive polyarticular JIA genetically appears to be a childhood‐onset presentation of RA supports further investigation of this phenotype along with the factors influencing an early‐onset presentation. Broadly, our results suggest that genetic profiling might enhance our ability to classify and understand the different phenotypes of inflammatory arthritis. Our results also provide a rationale for studying both diseases together and for translating therapeutic trials of successful pharmacologic agents from adult RA to RF‐positive polyarticular JIA and vice versa.

## Author contributions

All authors were involved in drafting the article or revising it critically for important intellectual content, and all authors approved the final version to be published. Dr. Prahalad had full access to all of the data in the study and takes responsibility for the integrity of the data and the accuracy of the data analysis.

### Study conception and design

Hinks, Marion, Cobb, Langefeld, Thompson, Thomson, Prahalad.

### Acquisition of data

Hinks, Marion, Cobb, Comeau, Sudman, Ainsworth, Bowes, Becker, Bohnsack, Haas, Lovell, Mellins, Nelson, Nordal, Punaro, Reed, Rose, Rosenberg, Rygg, Smith, Stevens, Videm, Wallace, Wedderburn, Yarwood, Yeung, Langefeld, Thompson, Thomson, Prahalad.

### Analysis and interpretation of data

Hinks, Marion, Cobb, Comeau, Sudman, Ainsworth, Bowes, Langefeld, Thompson, Thomson, Prahalad.
