# Supplementary material for: Brief Report: The Genetic Profile of Rheumatoid Factor–Positive Polyarticular Juvenile Idiopathic Arthritis Resembles That of Adult Rheumatoid Arthritis
Source: Arthritis Rheumatol. 2018 Apr 21;70(6):957–62. doi: 10.1002/art.40443 (PMC5984672; doi:10.1002/art.40443)
Supplement: Supplementary file 1 [file ART-70-957-s001.docx]

Supplementary Information

**Juvenile Arthritis Consortium for Immunochip (JACI) (In alphabetical order):** Hannah C. Ainsworth, Robert Andrews, Mara L. Becker, John F. Bohnsack, John Bowes, Matthew A. Brown, Milton Brown, Wei-Min Chen, Joanna Cobb, Patrick Concannon, Mary E. Comeau, Panos Deloukas, Sarah Edkins, Stephen Eyre, Patrick M. Gaffney, Stephen L. Guthery, Joel M. Guthridge, Johannes Peter Haas, Melissa Hazen, Anne Hinks, Sarah E. Hunt, Judith A. James, Carl D. Langefeld, Daniel J. Lovell, Miranda C. Marion, Kathy L. Moser, Peter A. Nigrovic, Ellen Nordal, Suna Onengut-Gumuscu, Sampath Prahalad, Marilyn Punaro, Carlos D. Rosé, Alan M. Rosenberg, Stephen S. Rich, Marite Rygg, Kathryn JA. Steel, Marc Sudman, Wendy Thomson, Susan D. Thompson, Vibeke Videm, Edward K. Wakeland, Carol A. Wallace, Lucy R. Wedderburn, Carol Wise, Patricia Woo, Rae SM. Yeung.

Biologically Based Outcome Predictors in JIA (BBOP)^#^, British Society of Paediatric and Adolescent Rheumatology (BSPAR) study group^#^, Childhood arthritis prospective study (CAPS)^#^, Childhood Arthritis Response to Medication Study (CHARMS)^#^, German society for Pediatric Rheumatology (GKJR)^#^, JIA gene expression studies^#^, NIAMS JIA genetic registry^#^, Nordic JIA study^#^, TREAT study^#^, Understanding TNF Therapy in JIA Project^#^, United Kingdom Juvenile Idiopathic Arthritis Genetics Consortium (UKJIAGC)^#^

**^#^Consortia**

**Biologically Based Outcome Predictors in JIA (BBOP):** Adam Baxter-Jones, Susanne Benseler, Sasha Bernatsky, Gilles Boire, David A Cabral, Bonnie Cameron, Sarah Campillo, Gaëlle Chédeville, Anne-Laure Chetaille, Paul Dancey, Joan Dietz, Ciaran Duffy, Karen Watanabe Duffy, Janet Ellsworth, Simon WM Eng, Brian Feldman, Sarah Finch, Marvin J Fritzler, Anna Goldenberg, John R Gordon, Jaime Guzman, David Hart, Daniel Hogan, Kristen Houghton, Adam Huber, Nicole Johnson, Roman Jurencak, Anthony Kusalik, Ron M Laxer, Loren Matheson, Deborah Levy, Bindu Nair, Kiem G Oen, Ross E Petty, Suzanne Ramsey, Elham Rezaei, Alan M Rosenberg, Johannes Roth, Dax Rumsey, Stephen W Scherer, Heinrike Schmeling, Rayfel Schneider, Rosie Scuccimarri, Elizabeth Stringer, Regina Taylor-Gjevre, Brett Trost, Shirley ML Tse, Lori B Tucker, Hassan Vatanparast, Richard F. Wintle, Rae SM Yeung.

**British Society of Paediatric and Adolescent Rheumatology (BSPAR) study group:** Mario Abinum, A. Bell, Alan W. Craft, Esther Crawley, Joel David, Helen Foster, Janet Gardener-Medwin, Jane Griffin, Ann Hall, M. Hall, Ariane L. Herrick, Peter Hollingworth, Lennox Holt, Stan Jones, Gillian Pountain, Clive Ryder, Tauny Southwood, I. Stewart, Helen Venning, Lucy R. Wedderburn, Patricia Woo, Sue Wyatt.

**Childhood Arthritis Prospective Study (CAPS):** Eileen Baildam, Nick Bishop, Lynsey Brown, Joanne Buckley, Alice Chieng, Roberto Carrasco, Joanna Cobb, Lucy Cook, Joyce Davidson, Annette Duggan, Michael Eltringham, Helen Foster, Elizabeth Friel, Mark Friswell, Janet Gardner-Medwin, Paul Gilbert, Vikki Gould, Kelly Hadfield, Kimme Hyrich, Julie Jones, Sham Lal, Mark Lay, Gabrielle Lloyd, Olivia Lloyd, Carol Lydon, Natasha Makengo, Ann McGovern, Alexandra Meijer, Nicola Mills-Wierda, Theresa Moorcroft, Vicki Price, Liang Qiao, Kay Riding, Jane Sim, Tauny Southwood, Wendy Thomson, Maureen Todd, Susan Tremble, Katharine Venter, Debbie Wade, Peter Ward, Sharon Watson, Gwen Webster, Lucy R Wedderburn, Jadranka Zelenovic.

**Childhood Arthritis Response to Medication Study (CHARMS)** Cherelle Allen, Kate Armon, Katrin Burkle, Joanna Cobb, Louise Coke, Catherine Cotter, Angela Etheridge, Edward Flynn, Elizabeth Fofana, Eirini Giannakopoulou, Paul Gilbert, Kimberley Gilmour, Clare Heard, Anne Hinks, Shashi Hirani, Ruth Howman, Laura Kassoumeri, Sham Lal, Alice Leahy, Lucy Marshall, Natasha Makengo, Laura Melville, Halima Moncrieffe, Kathleen Mulligan, Stanton Newman, Kiran Nistala, Jason Palman, Fiona Patrick, Emily Robinson, Stephanie Simou, Tauny Southwood, Stefanie Stafford, Balathas Thirugnanabalan, Wendy Thomson, Petra Tucker, Simona Ursu, Kishore Warrier, Joanna Watts, Lucy R. Wedderburn, Pamela Whitworth, Sadie Wickwar, Patricia Woo.

**German Society for Pediatric Rheumatology (GKJR):** Guenther Dannecker, Gerd Ganser Johannes Peter Haas, Hartmut Michels.

**JIA gene expression studies:** Mara L. Becker, Robert A. Colbert, Jason Dare, Beth S. Gottleib, Thomas A. Griffin, Alexi Grom, Norman T. Ilowite, Daniel J. Lovell, Halima Moncrieffe, Peter A. Nigrovic, Judy Ann Olsen, Sampath Prahalad, Margalit Rosenkranz, David D. Sherry, Susan D. Thompson.

**NIAMS JIA genetic registry:** John F. Bohnsack, Gloria Higgins, Marissa Klein-Gittelman, David N. Glass, T. Brent Graham, Thomas A. Griffin, Paula W. Morris, Natasha Ruth, Murray H. Passo, Sampath Prahalad, Stephen J Spalding, Susan D. Thompson.

**Nordic JIA study, Norwegian extended subcohort:** Marite Rygg, Ellen Nordal.

**The Nord-Trøndelag Health Study (HUNT):** Vibeke Videm, Matthew A. Brown.

**TREAT study:** Carol A. Wallace, Edward H. Giannini, Stephen J. Spalding, Phillip J. Hashkes, Kathleen M. O'Neil, Andrew S. Zeft, Illona S. Szer, Sarah Ringold, Hermine I. Brunner, Laura E. Schanberg, Robert P. Sundel, Diana Milojevic, Marilynn G. Punaro, Peter Chira, Beth S. Gottlieb, Gloria C. Higgins, Norman T. Ilowite, Yukiko Kimura, Stephanie Hamilton, Anne Johnson, Bin Huang, Daniel J. Lovell.

**Understanding TNF Therapy in JIA Project**: Daniel J. Lovell, Anne L. Johnson, Steven J. Spalding, Andrew Zeft, Beth S. Gottlieb, Paula W. Morris, Yukiko Kimura, Karen Onel, Suzanne C. Li, MD, Alexei A. Grom, Janalee Taylor, Hermine I. Brunner, Jennifer L. Huggins, James J. Nocton, Kathleen A. Haines, Barbara S. Edelheit, Michael Shishov, Lawrence K. Jung, Calvin B. Williams, Melissa S. Tesher, Denise M. Costanzo, Lawrence S. Zemel, Jason A. Dare, Murray H. Passo, Kaleo C. Ede, Judyann C. Olson, Elaine A. Cassidy, Thomas A. Griffin, Linda Wagner-Weiner, Jennifer E. Weiss, Sampath Prahalad, Larry B. Vogler, Kelly A. Rouster-Stevens, Timothy Beukelman, Randy Q. Cron, Daniel Kietz, Kara M. Schmidt, Kenneth Schikler, Jay Mehta, Tracy V. Ting, James W. Verbsky, Anne B. Eberhard, Bin Huang, Edward H. Giannini

**United Kingdom Juvenile Idiopathic Arthritis Genetics Consortium (UKJIAGC):** Eileen Baildam, Annette Bryant, Lindsay Cameron, Joyce Davidson, Rachelle Donn, Helen Foster, Paul Gilbert, Vikki Gould, Helen Hanson, Wheldon Houlsby, Emma Inness, Olivia Lloyd, Carol Lydon, Natasha Makengo, Alexandra Meijer, Jill Paxton, Liang Qiao, Kay Riding, Tauny Southwood, Wendy Thomson, Debbie Wade, Peter Ward, Lucy R Wedderburn, Nick Wilkinson, Patricia Woo.

Patient information

The post quality control (QC) United States (US) cohort comprised 222 US RF-positive polyarticular JIA patients and 4408 US controls. Clinics enrolling at least 5 JIA patients for Cincinnati-based studies (listed in order of number contributed) were located in Cincinnati, OH; Atlanta, GA; Columbus, OH; Little Rock, AR; Long Island, NY; Charlotte, NC; Cleveland, OH; Chicago, IL; Dover, DE; Nashville, TN; Philadelphia, PA; Milwaukee, WI; Toledo, OH; Charleston, SC; and Indianapolis, IN. Additional DNA from JIA cases collected independently by investigators in Salt Lake City, UT, Dallas, TX, Kansas City, MO and Boston, MA or enrolled as part of the Trial of Early Aggressive Therapy in Juvenile Idiopathic Arthritis (TREAT) study (clinical trials identifier NCT00443430) were made available for genotyping in Cincinnati.

The US controls were derived from four sources: healthy children without known major health conditions recruited from the geographical area served by Cincinnati Children’s Hospital Medical Center (CCHMC) and healthy adults collected at CCHMC. Healthy adult controls from Utah screened for autoimmune diseases and all were included in the replication cohort of previous GWAS studies[1;2]. Healthy adult controls collected at the Oklahoma Medical Research Foundation; and healthy US adult controls from the Genotype and Phenotype registry ([www.gapregistry.org](http://www.gapregistry.org)) and the NIDDK IBD Genetics Consortium. Healthy controls from the Oklahoma Medical Research Foundation (OMRF) were provided by the Lupus Family Registry and Repository (LFRR)[3] and the Oklahoma Immune Cohort (OIC). Each individual completed the Connective Tissue Disease Screening Questionnaire (CSQ)[4] and individuals with a “probable” systemic rheumatic disease were excluded. Each individual was enrolled into these studies after appropriate written consent and IRB approval by the OMRF and the University of Oklahoma Health Sciences Center. Healthy controls were also provided from the University of Minnesota SLE sibship collection[5] and these subjects were enrolled after appropriate written consent and IRB approval by the University of Minnesota. The US collections and their use in genetic studies have been approved by the Institutional Review Board of CCHMC and each collaborating center.

The post QC United Kingdom (UK) cohort comprised 94 RF-positive polyarticular JIA patients from five sources: The British Society for Paediatric and Adolescent Rheumatology (BSPAR) National Repository of JIA; a cohort of UK patients with long-standing JIA, described previously[6]; a cohort collected as part of the Childhood Arthritis Prospective Study (CAPS), a prospective inception cohort study of JIA cases from 5 centers across UK[7]; a cohort of children recruited for the SPARKS-CHARM (Childhood Arthritis Response to Medication) study, who fulfil ILAR criteria for JIA and are about to start new disease-modifying medication for active arthritis[8] and an ongoing collection of UK cases, the UK JIA Genetics Consortium (UKJIAGC). JIA cases were classified according to ILAR criteria[9]. There is overlap in the JIA cases used in this study and in previous UK candidate gene studies of JIA^[10-13]^. All UK JIA cases were recruited with ethical approval and provided informed consent [North-West Multi-Centre Research Ethics Committee (MREC 99/8/84), the University of Manchester Committee on the Ethics of Research on Human Beings and National Research Ethics Service (NRES 02/8/104)]. The UK controls comprised the shared UK 1958 Birth cohort and UK Blood Services Common Controls. The collection was established as part of the WTCCC[14].

The post QC German cohort comprised 1 German RF-positive polyarticular JIA patient and 480 controls. These cases have already been included as a replication cohort in a genome-wide association study and previously described. These patients were recruited from the German Center for Rheumatology in Children and Adolescents, Garmisch-Partenkirchen; the Department of Pediatrics, University of Tübingen; Children's Rheumatology Unit Sendenhorst, Germany; and the Department of Pediatrics, University of Prague, Czech Republic. JIA was determined retrospectively by chart review. German population-based control samples were prepared from cord blood obtained from healthy newborns in the Survey of Neonates in Pomerania (SNiP) consortium[15]. The respective Institutional Review Boards approved the collection of these samples and participation in this study.

The post QC Norwegian cohort comprised 13 Norwegian RF-positive polyarticular JIA patients and 945 controls. The patient cohort comprised consecutive cases of JIA from two defined geographical areas in Norway with disease onset from 2000-2012 and with available DNA samples. The study was an extended part of the Nordic JIA Study, a prospective, multicentre cohort study initiated by NoSPeR (Nordic Study group of Pediatric Rheumatology) as previously described[16]. The cohort aimed to be as close to population-based as possible, as centres participated only if they were able to include all children diagnosed with JIA in their catchment area. Approval from medical and health research ethical committees and data authorities were granted and informed consent obtained from parents and/or children according to national regulations. The Norwegian controls were from the second (1995-1997) or third (2006-2008) waves of the population-based Nord-Trøndelag Health Study (HUNT) ([www.ntnu.edu/hunt](http://www.ntnu.edu/hunt)). They were a random selection of participants who had answered “no” to questions of whether they had ever been given a diagnosis of rheumatoid arthritis or ankylosing spondylitis. All HUNT participants gave written, informed consent. The study was approved by the Regional Committee for Medical and Health Research Ethics, Central Norway, the Norwegian Data Inspectorate, and the Norwegian Department of Health.

The post QC Canadian Cohort comprised 10 Canadian RF-positive polyarticular JIA patients. The BBOP study (Biologically Based Outcome Predictors in JIA) is a multi-center inception cohort of prospectively collected children with new onset JIA recruited from 11 Canadian Pediatric Rheumatology Programs. Children were included in the study if they satisfied the ILAR classification criteria, were within six months of disease onset, and were treatment-naïve for medications except for non-steroidal anti-inflammatory agents. Peripheral blood and saliva samples were collected and processed as per standardized protocol at enrolment and transported to the central biobank (The Hospital for Sick Children (SickKids), Toronto, ON). Informed consent for participation was obtained from parents and informed consent or assent was obtained from patients as applicable. The study was approved by the Research Ethics Board at each participating site.

**Supplementary Table 1 Samples used in the wGRS analyses**

|  | **Cases** | **Controls** |
| --- | --- | --- |
| **RF-positive polyarticular JIA** | Total 340  UK=94  US=222  Germany=1  Norway=13  Canada=10 | Total 10147  UK=4337*  US=4386  Germany=480  Norway=945 |
| **Early-onset RA (16-29 yrs)** | UK=370 | UK=4338^#^ |
| **Later-onset RA (≥70 yrs)** | UK=259 | UK=4338^#^ |

*These 4337 UK controls are unique compared to the ^#^4338 used in the RA analysis.

**Supplementary Table 2 Association results in RF-positive polyarticular JIA of the 27 non-HLA SNPs most strongly associated with RF-negative polyarticular and oligoarticular JIA**

| **SNP** | **Chr** | **Position** | **Gene Region** | **RF-poJIA minor allele** | **RF-poJIA risk allele** | **reference allele** | **MAF RF-poJIA** | **MAF controls** | **MAF RF+pJIA** | **RF-poJIA OR** | **RF-poJIA CI** | **best p-value RF+pJIA** | **Model** | **RF+pJIA OR** | **RF+pJIA CI** |
| --- | --- | --- | --- | --- | --- | --- | --- | --- | --- | --- | --- | --- | --- | --- | --- |
| rs4648881 | 1 | 25197155 | *RUNX3* | G | G | G | 0.47 | 0.49 | 0.51 | 1.16 | 1.1-1.23 | 0.91 | add | 1.01 | 0.87-1.18 |
| **rs6679677** | **1** | **114303808** | ***PTPN22*** | **A** | **A** | **A** | **0.14** | **0.10** | **0.15** | **1.59** | **1.45-1.73** | **1.21E-06** | **dom** | **1.83** | **1.43-2.33** |
| rs11265608 | 1 | 154364140 | *ATP8B2 \| IL6R* | A | A | A | 0.12 | 0.10 | 0.12 | 1.28 | 1.16-1.4 | 0.17 | add | 1.18 | 0.93-1.49 |
| rs6740838 | 2 | 100813499 | *AFF3 \| LONRF2* | A | A | A | 0.43 | 0.39 | 0.42 | 1.25 | 1.14-1.37 | 0.20 | add | 1.11 | 0.95-1.29 |
| rs10174238 | 2 | 191973034 | *STAT4* | G | G | G | 0.28 | 0.23 | 0.27 | 1.29 | 1.2-1.37 | 0.08 | add | 1.17 | 0.98-1.39 |
| **rs79893749** | **3** | **46253650** | ***CCR1 \| CCR3*** | **A** | **G** | **A** | **0.12** | **0.15** | **0.11** | **0.79** | **0.72-0.86** | **0.009** | **add** | **0.72** | **0.56-0.92** |
| rs4688013 | 3 | 119229486 | *TIMMDC1 \| CD80* | A | A | A | 0.22 | 0.19 | 0.17 | 1.20 | 1.12-1.29 | 0.16 | add | 0.86 | 0.7-1.06 |
| rs1479924 | 4 | 123387600 | *IL2 \| IL21* | G | A | G | 0.24 | 0.29 | 0.29 | 0.80 | 0.74-0.85 | 0.91 | add | 1.01 | 0.85-1.2 |
| **rs71624119** | **5** | **55440730** | ***ANKRD55*** | **A** | **G** | **A** | **0.20** | **0.25** | **0.19** | **0.78** | **0.73-0.84** | **0.003** | **add** | **0.74** | **0.61-0.9** |
| rs27290 | 5 | 96350088 | *ERAP2 \| LNPEP* | G | G | G | 0.47 | 0.44 | 0.43 | 1.32 | 1.2-1.45 | 0.98 | add | 1.00 | 0.86-1.16 |
| rs4705862 | 5 | 131813219 | *C5orf56 \| IRF1* | T | A | T | 0.39 | 0.44 | 0.43 | 0.84 | 0.79-0.89 | 0.78 | add | 0.98 | 0.84-1.14 |
| rs7808122 | 7 | 22798080 | *IL6* | A | A | A | 0.48 | 0.44 | 0.41 | 1.18 | 1.11-1.25 | 0.46 | add | 0.94 | 0.81-1.1 |
| rs10280937 | 7 | 28182306 | *JAZF1* | G | G | G | 0.13 | 0.11 | 0.11 | 1.25 | 1.14-1.37 | 0.82 | add | 0.97 | 0.76-1.24 |
| rs7909519 | 10 | 6089841 | *IL2RA* | C | A | C | 0.08 | 0.11 | 0.08 | 0.72 | 0.64-0.8 | 0.08 | add | 0.78 | 0.6-1.03 |
| rs7069750 | 10 | 90762376 | *FAS* | C | C | C | 0.48 | 0.44 | 0.43 | 1.18 | 1.11-1.25 | 0.74 | add | 0.97 | 0.83-1.14 |
| rs4755450 | 11 | 36363575 | *PRR5L* | A | G | A | 0.31 | 0.35 | 0.33 | 0.85 | 0.8-0.91 | 0.37 | add | 0.93 | 0.79-1.09 |
| rs2364480 | 12 | 6495275 | *LTBR* | C | C | C | 0.28 | 0.25 | 0.24 | 1.20 | 1.12-1.28 | 0.31 | add | 0.91 | 0.76-1.09 |
| **rs3184504** | **12** | **111884608** | ***SH2B3 \| ATXN2*** | **A** | **A** | **A** | **0.54** | **0.49** | **0.53** | **1.20** | **1.13-1.27** | **0.01** | **add** | **1.22** | **1.04-1.42** |
| rs7993214 | 13 | 40350912 | *COG6* | A | G | A | 0.31 | 0.35 | 0.32 | 0.84 | 0.79-0.9 | 0.23 | add | 0.91 | 0.77-1.07 |
| rs34132030 | 13 | 43056036 | *13q14* | A | A | A | 0.32 | 0.29 | 0.31 | 1.18 | 1.11-1.26 | 0.13 | add | 1.14 | 0.96-1.34 |
| rs12434551 | 14 | 69253364 | *ZFP36L1* | A | T | A | 0.43 | 0.47 | 0.45 | 0.85 | 0.8-0.9 | 0.30 | add | 0.92 | 0.79-1.07 |
| **rs66718203** | **16** | **11428643** | ***PRM1 \| RMI2*** | **C** | **G** | **C** | **0.14** | **0.18** | **0.14** | **0.81** | **0.74-0.88** | **0.006** | **add** | **0.73** | **0.58-0.91** |
| rs2847293 | 18 | 12782448 | *PTPN2* | A | A | A | 0.20 | 0.17 | 0.18 | 1.31 | 1.22-1.4 | 0.32 | add | 1.11 | 0.91-1.36 |
| **rs34536443** | **19** | **10463118** | ***TYK2*** | **G** | **C** | **G** | **0.03** | **0.05** | **0.02** | **0.56** | **0.47-0.67** | **0.01** | **add** | **0.53** | **0.33-0.88** |
| rs9979383 | 21 | 36715761 | *RUNX1* | G | A | G | 0.33 | 0.37 | 0.39 | 0.78 | 0.72-0.85 | 0.37 | add | 1.07 | 0.92-1.26 |
| rs2266959 | 22 | 21922904 | *UBE2L3* | A | A | A | 0.22 | 0.19 | 0.21 | 1.24 | 1.15-1.33 | 0.41 | add | 1.08 | 0.9-1.31 |
| rs2284033 | 22 | 37534034 | *IL2RB* | A | G | A | 0.39 | 0.44 | 0.43 | 0.84 | 0.79-0.89 | 0.70 | add | 0.97 | 0.83-1.13 |

RF-poJIA=RF-negative polyarticular and oligoarticular JIA, RF+pJIA=RF-positive polyarticular JIA, Chr=chromosome, SNP=single nucleotide polymorphism, MAF=minor allele frequency, dom=dominant, add=additive, OR=odds ratio, CI=confidence interval. Regions with p-value <0.05 are highlighted in bold.

**Supplementary Table 3 Association results in RF-positive polyarticular JIA of the 44 non-HLA SNPs most strongly associated with RA**

| **SNP** | **Chr** | **Position** | **Gene region** | **RA risk allele** | **RF+ pJIA ref allele** | **MAF RF+ pJIA** | **MAF controls** | **RA OR** | **RF+ pJIA best pvalue** | **Model** | **OR** | **CI** |
| --- | --- | --- | --- | --- | --- | --- | --- | --- | --- | --- | --- | --- |
| **rs2843401** | **1** | **2517993** | ***MMEL1*** | **A** | **A** | **0.27** | **0.33** | **0.87** | **5.05E-04** | **add** | **0.74** | **0.62-0.87** |
| **rs2240336** | **1** | **17546989** | ***PADI4*** | **A** | **A** | **0.38** | **0.42** | **0.88** | **1.96E-02** | **add** | **0.83** | **0.71-0.97** |
| rs883220 | 1 | 38389458 | *POU3F1* | A | A | 0.24 | 0.25 | 0.89 | 6.05E-01 | add | 0.95 | 0.8-1.14 |
| **rs2476601** | **1** | **114179091** | ***PTPN22*** | **A** | **A** | **0.15** | **0.1** | **1.78** | **1.30E-06** | **dom** | **1.83** | **1.43-2.33** |
| rs798000 | 1 | 117082219 | *CD2* | G | G | 0.33 | 0.32 | 1.11 | 3.85E-01 | add | 1.07 | 0.91-1.26 |
| **rs8192284** | **1** | **152693594** | ***IL6R*** | **C** | **C** | **0.36** | **0.41** | **0.9** | **1.68E-02** | **add** | **0.82** | **0.7-0.97** |
| rs10494360 | 1 | 159742374 | *FCGR2A* | G | A | 0.11 | 0.12 | 1.14 | 2.48E-01 | add | 0.86 | 0.67-1.11 |
| rs2014863 | 1 | 197076224 | *PTPRC* | C | G | 0.38 | 0.37 | 1.09 | 4.77E-01 | add | 1.06 | 0.9-1.24 |
| rs34695944 | 2 | 60978354 | *REL* | G | G | 0.37 | 0.37 | 1.13 | 4.30E-01 | add | 1.07 | 0.91-1.25 |
| rs6546146 | 2 | 65409828 | *SPRED2* | A | A | 0.37 | 0.37 | 0.9 | 7.49E-01 | add | 0.97 | 0.83-1.14 |
| rs10209110 | 2 | 100039124 | *AFF3* | A | A | 0.49 | 0.5 | 0.9 | 6.46E-01 | add | 0.96 | 0.83-1.12 |
| rs13426947 | 2 | 191641499 | *STAT4* | A | A | 0.22 | 0.19 | 1.15 | 9.22E-02 | add | 1.17 | 0.97-1.42 |
| **rs11571302** | **2** | **204451179** | ***CTLA4*** | **A** | **A** | **0.43** | **0.47** | **0.89** | **2.41E-02** | **add** | **0.84** | **0.72-0.98** |
| **rs1980422** | **2** | **204318641** | ***CD28*** | **G** | **G** | **0.27** | **0.23** | **1.12** | **2.36E-02** | **add** | **1.22** | **1.03-1.45** |
| rs35677470 | 3 | 58158676 | *DNASE1L3* | A | A | 0.08 | 0.08 | 1.19 | 9.51E-01 | add | 1.01 | 0.76-1.35 |
| **rs932036** | **4** | **25699960** | ***RBPJ*** | **A** | **T** | **0.33** | **0.3** | **1.14** | **4.29E-02** | **add** | **1.18** | **1.01-1.39** |
| rs78560100 | 4 | 123260921 | *IL2-IL21* | C | C | 0.08 | 0.07 | 1.13 | 3.94E-01 | add | 1.13 | 0.85-1.5 |
| **rs71624119** | **5** | **55476487** | ***ANKRD55*** | **A** | **A** | **0.19** | **0.24** | **0.81** | **2.91E-03** | **add** | **0.74** | **0.61-0.9** |
| rs39984 | 5 | 102625191 | *GIN1* | A | A | 0.29 | 0.32 | 0.88 | 1.15E-01 | add | 0.87 | 0.73-1.03 |
| rs6911690 | 6 | 106577656 | *PRDM1* | G | G | 0.11 | 0.11 | 0.87 | 8.16E-01 | add | 0.97 | 0.76-1.24 |
| **rs6920220** | **6** | **138048197** | ***TNFAIP3*** | **A** | **A** | **0.25** | **0.22** | **1.2** | **4.42E-03** | **add** | **1.29** | **1.08-1.54** |
| rs629326 | 6 | 159416701 | *TAGAP* | C | C | 0.4 | 0.41 | 0.9 | 4.65E-01 | add | 0.94 | 0.81-1.1 |
| **rs59466457** | **6** | **167457744** | ***CCR6*** | **A** | **A** | **0.51** | **0.44** | **1.15** | **4.37E-04** | **add** | **1.32** | **1.13-1.54** |
| **rs3807306** | **7** | **128367916** | ***IRF5*** | **C** | **A** | **0.56** | **0.49** | **0.89** | **6.46E-04** | **add** | **1.31** | **1.12-1.53** |
| **rs4840565** | **8** | **11382954** | ***BLK*** | **G** | **G** | **0.34** | **0.27** | **1.1** | **5.31E-04** | **add** | **1.33** | **1.13-1.57** |
| rs2812378 | 9 | 34700260 | *CCL21* | G | G | 0.38 | 0.34 | 1.15 | 5.87E-02 | add | 1.16 | 0.99-1.36 |
| rs10739580 | 9 | 122735103 | *TRAF1* | G | G | 0.35 | 0.35 | 1.12 | 8.86E-01 | add | 1.01 | 0.86-1.19 |
| **rs10795791** | **10** | **6148346** | ***IL2RA*** | **G** | **G** | **0.46** | **0.41** | **1.09** | **2.02E-02** | **add** | **1.2** | **1.03-1.4** |
| rs947474 | 10 | 6430456 | *PRKCQ* | G | G | 0.18 | 0.19 | 0.9 | 9.14E-01 | add | 0.99 | 0.81-1.2 |
| **rs2275806** | **10** | **8135346** | ***GATA3*** | **G** | **G** | **0.48** | **0.43** | **1.11** | **2.36E-02** | **add** | **1.19** | **1.02-1.39** |
| rs12764378 | 10 | 63470010 | *ARID5B* | A | A | 0.25 | 0.22 | 1.14 | 1.06E-01 | add | 1.16 | 0.97-1.38 |
| rs570676 | 11 | 36448767 | *TRAF6* | A | A | 0.38 | 0.39 | 0.93 | 4.80E-01 | add | 0.94 | 0.81-1.11 |
| rs595158 | 11 | 60666157 | *CD5* | C | A | 0.51 | 0.5 | 1.09 | 6.51E-01 | add | 1.04 | 0.89-1.21 |
| **rs4938573** | **11** | **118247052** | ***DDX6*** | **G** | **G** | **0.15** | **0.19** | **0.87** | **3.44E-03** | **add** | **0.72** | **0.58-0.9** |
| rs8043085 | 15 | 36615432 | *RASGRP1* | A | A | 0.23 | 0.24 | 1.17 | 3.67E-01 | add | 0.92 | 0.77-1.1 |
| **rs8026898** | **15** | **67778471** | ***TLE3*** | **A** | **A** | **0.31** | **0.27** | **1.17** | **2.09E-02** | **add** | **1.22** | **1.03-1.44** |
| rs13330176 | 16 | 84576588 | *IRF8* | A | A | 0.27 | 0.23 | 1.15 | 7.74E-02 | add | 1.17 | 0.98-1.39 |
| rs12936409 | 17 | 35297175 | *IKZF3* | A | A | 0.48 | 0.47 | 1.1 | 2.54E-01 | add | 1.09 | 0.94-1.28 |
| **rs34536443** | **19** | **10324118** | ***TYK2*** | **G** | **G** | **0.02** | **0.05** | **0.62** | **1.36E-02** | **add** | **0.53** | **0.33-0.88** |
| rs6032662 | 20 | 44167717 | *CD40* | G | G | 0.23 | 0.25 | 0.86 | 4.28E-01 | add | 0.93 | 0.77-1.12 |
| rs2834512 | 21 | 34833469 | *RCAN1* | A | A | 0.11 | 0.12 | 0.86 | 2.58E-01 | add | 0.87 | 0.68-1.11 |
| rs9979383 | 21 | 35637631 | *RUNX1* | G | G | 0.39 | 0.37 | 0.9 | 3.71E-01 | add | 1.07 | 0.92-1.26 |
| **rs3218251** | **22** | **35875451** | ***IL2RB*** | **A** | **A** | **0.29** | **0.26** | **1.13** | **3.88E-02** | **add** | **1.19** | **1.01-1.41** |
| **rs13397*** | **23** | **153248248** | ***IRAK1*** | **A** | **A** | **0.18** | **0.12** | **1.27** | **1.49E-02** | **add** | **1.37** |  |

This list does not include *HLA* and *KIF5A* region, the latter is a deletion polymorphism and not analysed in this study.

RF-poJIA=RF-negative polyarticular and oligoarticular JIA, RF+pJIA=RF-positive polyarticular JIA, Chr=chromosome, SNP=single nucleotide polymorphism, MAF=minor allele frequency, dom=dominant, add=additive, OR=odds ratio, CI=confidence interval.

* MAF for this SNP is for females only. The p-value and OR are from the meta-analysis of females and male cohorts. Regions with p-value <0.05 are highlighted in bold.

**Supplementary Table 4 Most significant associations in RF-positive polyarticular JIA**

| Gene Region | Most Significant  SNP | Chr | Position^a^ | Ref.  Allele | RAF  Cases | RAF  Controls | *P* value | Model | OR (95% CI) | SNP Location |
| --- | --- | --- | --- | --- | --- | --- | --- | --- | --- | --- |
| *MMEL1-TTC34* | rs7526311 | 1 | 2697298 | T | 0.50 | 0.43 | 1.21 x 10^-5^ | Dominant | 1.81 (1.39-2.35) | INTERGENIC |
| *PTPN22* | rs6679677 | 1 | 114303808 | A | 0.15 | 0.10 | 1.21 x 10^-6^ | Dominant | 1.83 (1.43-2.33) | INTERGENIC |
| *PTGS2* | rs6681231 | 1 | 186659859 | C | 0.22 | 0.16 | 1.60 x 10^-5^ | Additive | 1.51 (1.25-1.81) | INTERGENIC |
| *PTGS2* | rs20432^b^ | 1 | 186646323 | C | 0.21 | 0.15 | 7.13 x 10^-6^ | Additive | 1.63 (1.32-2.02) | INTRON |
| *FAM19A1* | rs7617075 | 3 | 68240106 | T | 0.14 | 0.20 | 4.09 x 10^-5^ | Dominant | 0.59 (0.46-0.76) | INTRON |
| *PABPC4L* | rs970036 | 4 | 135040295 | G | 0.47 | 0.43 | 4.59 x 10^-6^ | Recessive | 1.78 (1.39-2.28) | INTERGENIC |
| *THEMIS* | rs62425513 | 6 | 128077738 | T | 0.22 | 0.16 | 5.03 x 10^-5^ | Additive | 1.47 (1.22-1.78) | INTRON |
| *LOC100130476-TNFAIP3* | rs610366 | 6 | 138139694 | G | 0.09 | 0.15 | 7.52 x 10^-5^ | Dominant | 0.56 (0.42-0.75) | INTERGENIC |
| *IRF5-TNPO3* | rs4731531 | 7 | 128562647 | A | 0.54 | 0.46 | 2.60 x 10^-5^ | Additive | 1.39 (1.19-1.62) | INTERGENIC |
| *XKR6 - BLK* | rs9657519 | 8 | 10945439 | A | 0.56 | 0.49 | 5.89 x 10^-5^ | Recessive | 1.60 (1.27-2.02) | INTRON (XKR6) |
| *SERGEF* | rs2158395 | 11 | 17828930 | G | 0.27 | 0.35 | 3.59 x 10^-5^ | Additive | 0.70 (0.59-0.83) | INTRON |
| *SERGEF* | rs4757590^b^ | 11 | 17822569 | C | 0.26 | 0.34 | 2.23 x 10^-5^ | Additive | 0.70 (0.60-0.83) | INTRON |
| *OR9G4-LOC101927120* | rs11228824 | 11 | 56634316 | A | 0.24 | 0.19 | 6.50 x 10^-5^ | Additive | 1.45 (1.21-1.73) | INTERGENIC |
| *SHMT2* | rs2229717 | 12 | 57627074 | T | 0.06 | 0.03 | 7.98 x 10^-5^ | Dominant | 1.99 (1.42-2.81) | SYNONYMOUS CODING |
| *SHMT2* | rs28365862^b^ | 12 | 57623363 | G | 0.07 | 0.04 | 3.92 x 10^-5^ | Additive | 2.38 (1.58-3.61) | INTERGENIC |
| *RAC2* | rs9610687 | 22 | 37642869 | A | 0.25 | 0.34 | 4.14 x 10^-7^ | Dominant | 0.57 (0.45-0.71) | INTERGENIC |

Chr., chromosome; Ref., reference; RAF, reference allele frequency; OR, odds ratio; CI, confidence interval; FDR, false discovery rate.

^a^Coordinates are based on the GRCh37 assembly. ^b^Imputed SNP results are included when the imputed ^b^SNP had a better imputed *P* value than the most significant directly genotyped SNP in the region.

**Supplementary Figure 1 Manhattan plot of association statistics for RF-positive polyarticular JIA risk loci**


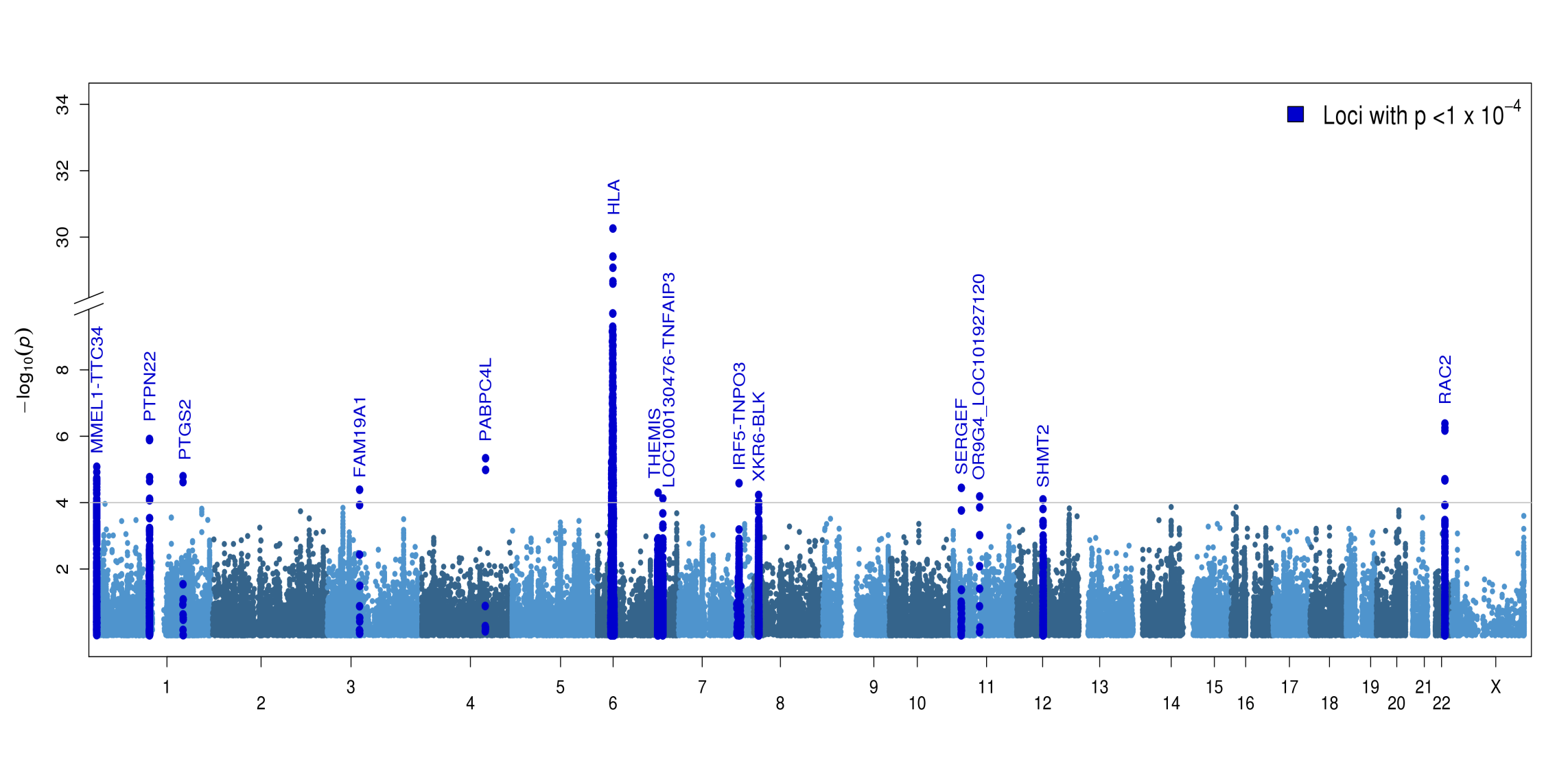


**Supplementary Figure 2 Regional association plots for the most significant associations in RF-positive polyarticular JIA**

**Regional association plots, generated using LocusZoom, for the 13 regions associated with JIA (P<1 x 10^-4^) on the left x axis is the –log10 p-value for the SNPs across the region, on the right x axis is the recombination rate across the region and on the y axis is the chromosomal position (Mb). Coordinates are based on the NCBI37 assembly. The SNPs that are genotyped are represented by circles, the SNPs that are imputed by squares. The dots/squares are coloured according to LD (r^2^) with the top SNP.**

Reference List

(1) Thompson SD, Sudman M, Ramos PS, et al. The susceptibility loci juvenile idiopathic arthritis shares with other autoimmune diseases extend to PTPN2, COG6, and ANGPT1. *Arthritis Rheum* 2010 Nov;62**:**3265-76.

(2) Thompson SD, Marion MC, Sudman M, et al. Genome-wide association analysis of juvenile idiopathic arthritis identifies a new susceptibility locus at chromosomal region 3q13. *Arthritis Rheum* 2012 Feb 21.

(3) Rasmussen A, Sevier S, Kelly JA, et al. The lupus family registry and repository. *Rheumatology (Oxford)* 2011 Jan;50**:**47-59.

(4) Karlson EW, Sanchez-Guerrero J, Wright EA, et al. A connective tissue disease screening questionnaire for population studies. *Ann Epidemiol* 1995 Jul;5**:**297-302.

(5) Gaffney PM, Ortmann WA, Selby SA, et al. Genome screening in human systemic lupus erythematosus: Results from a second Minnesota Cohort and combined analyses of 187 sib-pair families. *American journal of Human Genetics* 2000;66**:**547-56.

(6) Packham JC, Hall MA. Long-term follow-up of 246 adults with juvenile idiopathic arthritis: functional outcome. *Rheumatology (Oxford)* 2002 Dec;41**:**1428-35.

(7) Adib N, Hyrich K, Thornton J, et al. Association between duration of symptoms and severity of disease at first presentation to paediatric rheumatology: results from the Childhood Arthritis Prospective Study. *Rheumatology (Oxford)* 2008 Jul;47**:**991-5.

(8) Moncrieffe H, Hinks A, Ursu S, et al. Generation of novel pharmacogenomic candidates in response to methotrexate in juvenile idiopathic arthritis: correlation between gene expression and genotype. *Pharmacogenet Genomics* 2010 Nov;20**:**665-76.

(9) Petty RE, Southwood TR, Manners P, et al. International League of Associations for Rheumatology classification of juvenile idiopathic arthritis: second revision, Edmonton, 2001. *J Rheumatol* 2004 Feb;31**:**390-2.

(10) Hinks A, Ke X, Barton A, et al. Association of the IL2RA/CD25 gene with juvenile idiopathic arthritis. *Arthritis Rheum* 2009 Jan;60**:**251-7.

(11) Hinks A, Martin P, Flynn E, et al. Association of the CCR5 gene with juvenile idiopathic arthritis. *Genes Immun* 2010 Oct;11**:**584-9.

(12) Hinks A, Eyre S, Ke X, et al. Association of the AFF3 gene and IL2/IL21 gene region with juvenile idiopathic arthritis. *Genes Immun* 2010 Mar;11**:**194-8.

(13) Hinks A, Eyre S, Ke X, et al. Overlap of disease susceptibility loci for rheumatoid arthritis and juvenile idiopathic arthritis. *Ann Rheum Dis* 2010 Jun;69**:**1049-53.

(14) The Wellcome Trust Case Control consortium. Genome-wide association study of 14,000 cases of seven common diseases and 3,000 shared controls. *Nature* 2007 Jun 7;447**:**661-78.

(15) Beyersdorff A, Hoffmann W, Lingnau ML, et al. Survey of Neonates in Pomerania (SniP): a population based analysis of the mothers' quality of life after delivery with special relations to their social integration. *Int J Public Health* 2008;53**:**87-95.

(16) Nordal E, Zak M, Aalto K, et al. Ongoing disease activity and changing categories in a long-term nordic cohort study of juvenile idiopathic arthritis. *Arthritis Rheum* 2011 Sep;63**:**2809-18.

(17) Hinks A, Cobb J, Marion MC, et al. Dense genotyping of immune-related disease regions identifies 14 new susceptibility loci for juvenile idiopathic arthritis. *Nat Genet* 2013 Jun;45**:**664-9.
